# Quantitative risk analysis using vulnerability indicators to assess food insecurity in the Niayes agricultural region of West Senegal

**DOI:** 10.4102/jamba.v9i1.379

**Published:** 2017-11-30

**Authors:** Mateugue Diack, Macoumba Loum, Cheikh T. Diop, Ailsa Holloway

**Affiliations:** 1 UFR des Sciences Agronomiques, de l’Aquaculture et des Technologies Alimentaires, Université Gaston Berger, Sénégal; 2 UFR des Sciences Appliquées et Technologies, Université Gaston Berger, Sénégal; 3 Research Alliance for Disaster and Risk Reduction, Stellenbosch University, South Africa

## Abstract

There is an increasing need to develop indicators of vulnerability and adaptive capacity to determine the robustness of response strategies over time and better understand the underlying processes. This study aimed to determine levels of risk of food insecurity using defined vulnerability indicators. For the purpose of this study, factors influencing food insecurity and different vulnerable indicators were examined using quantitative and qualitative research methods. Observations made on the physical environment (using tools for spatial analysis) and socio-economic surveys conducted with local populations have quantified vulnerability indicators in the Niayes agricultural region. Application of the Classification and Regression Tree (CART) model has enabled us to quantify the level of vulnerability of the zone. The results show that the decrease in agricultural surface areas is the most discriminant one in this study. The speed of reduction of the agricultural areas has specially increased between 2009 and 2014, with a loss of 65% of these areas. Therefore, a decision-making system, centred on the need for reinforcing the resilience of local populations, by preserving the agricultural vocation of the Niayes region and even in the Sahelian regions requires support and extension services for the farmers in order to promote sustainable agricultural practices.

## Introduction

**FIGURE 1:**
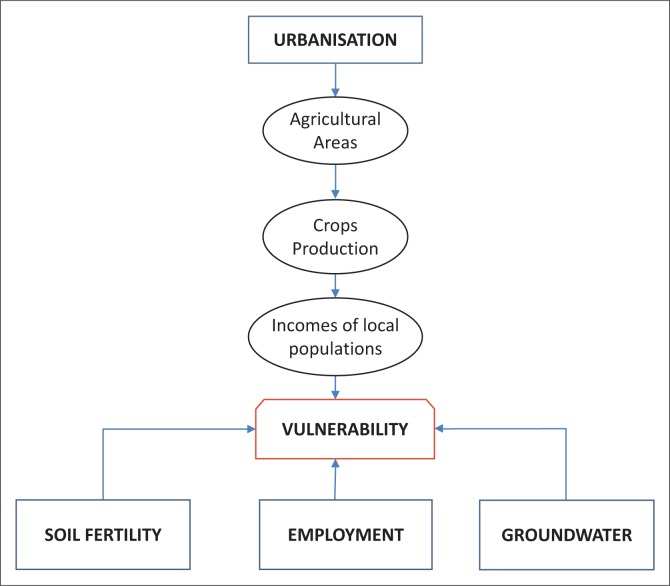
Conceptual model of analysis of the vulnerability in the Niayes region.

Vulnerability, hazard, adaptation or resilience are key concepts used in risk analysis (Brooks 2003). A good understanding of the vulnerability level of a community or a region using data that are especially quantitative can enable the development of strategies for adequate response to risk for food insecurity. Within the framework of a spatial analysis, food security can be apprehended at local, regional, national or even global scale, whereas a sociologic approach may be interested in the households’ issues (Leroy et al. 2015; Smith, Alderman & Aduayom 2006; Villagran de Leon 2006). In addition to these approaches, methods of quantitative analysis, including statistical indices, are tested through global risk analysis (Alinovi et al. 2010; Ligon & Schechter 2003). Regression trees are also calibrated for a quantitative validation of the data analysis on risks (Alinovi et al. 2009; Yohannes & Webb 1999). Such diversity of approaches and methods of analysis underlines existing difficulties to characterise variables used for risk analysis because they are rarely accessible through direct measurement (Allen 2003; Downing et al. 2001; Webb et al. 2006). In the Sahelian regions, the fragile capacity of adaptation of the populations to food insecurity and the challenge to reinforce the resilience of these populations pose the need to validate a method of analysis of the vulnerability that is reproducible to different agricultural production systems. Vulnerability is a key concept used to describe the state of susceptibility of a social or ecologic system facing disaster risks (Adger 2006). Food and nutritional insecurity is a key dimension of the vulnerability, mainly for the households under poverty (Kanbur 2014). Dynamics of the rural areas of Senegal are emphasised by agricultural production systems that are exposed to climatic change effects and anthropic pressure. At the moment where agricultural lands, including cropping and grazing areas, gradually increase with the detriment of the forestry reserves (Ahmed et al. 2015), urbanisation is leading to a reduction of arable lands (Sy et al. 2014). In the Dakar region, for example, urban population has increased from 88.4% in 1976 to 97.2% in 2008 (ANSD 2009). Taking into account the contribution from agricultural resources in expressing the need for subsistence of the populations, food security in the Sahelian countries is largely threatened. The estimate of global indices for hunger at the world scale has shown the highest scores for sub-Saharan Africa (18.2), followed by countries from South Asia with a score of 18.1 (IFPRI 2014). Projections on the food request in these sub-Saharan countries are also predicting a decrease of 35 millions of tons of cereals by 2025 (Cooper et al. 2008). This study is contributing to the quantitative approach of a risk analysis in general and a characterisation of vulnerability in particular (Brooks et al. 2005). It is applied in the Niayes region, which is exposed to risk factors related to urbanisation, industrialisation and installation of public works (Figure 1). In this region, agricultural areas are decreasing with the profit of human settlements and industrial companies (Diack et al. 2017). Such dynamics of occupation of the soils pose risks of vulnerability for food insecurity while threatening the disappearance of farming activities despite a high potential of soil fertility and an availability of water resources (Giradin et al. 1999; Luers et al. 2003). Thus, to assess the vulnerability of food insecurity, the Classification and Regression Tree (CART) model, which is an algorithm, is applied in this quantitative study. Agronomic and socio-economic variables of the area have been used as data for calibrating the model (Figure 1).

## Materials and methods

### The study area

**FIGURE 2:**
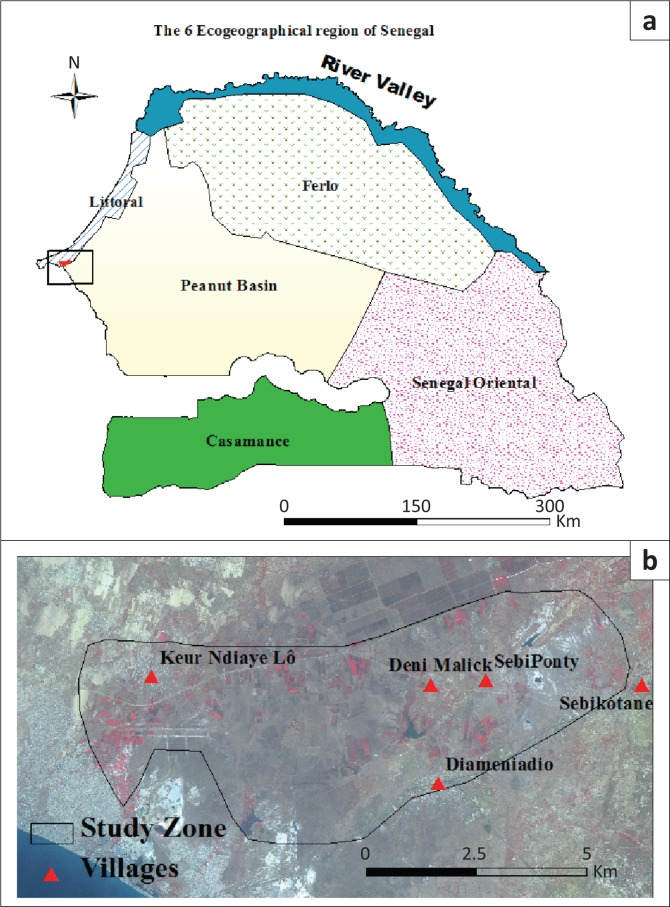
Localisation of the study area. (a) Agro-ecological zones and (b) villages of the Niayes region.

The Niayes region is the eco-geographical area of the Senegalese coast, of about 180 km long and 25 km wide. It is bounded to the west by the Atlantic Ocean and to the east by the national road of Dakar-Saint Louis. The land of this region is suitable for farming with a good soil quality level and available water resources because of a shallow groundwater. Market gardening, arboriculture and fishing are the main socio-economic activities. Until 2004, the Niayes region provided 60% of consumption needs of the national vegetable production and simultaneously 80% of the exports of horticultural products (Toure & Seck 2005). The richness of the biodiversity and the proximity to the sea give to the Niayes area a microclimate with more moderate temperatures compared to the climate in the interior regions. One of the consequences of these moderate biophysical conditions was an obvious change in the land use in the Niayes region. This study on vulnerability indicators was applied in a set of reference of 4360 ha land including the villages of Sébikotane, SébiPonty, Deni Malick Gueye and Keur Ndiaye Lo (Figure 2). The climate presents oceanic features. The maritime trade winds and ocean currents mitigate seasonal thermal contrasts of the Sahelian climate (Aguiar 2009). The rainy season lasts 3 months (July–October). The temperatures are recorded with a maximum of 30 °C. In cool dry season (December through February), the minima can decrease to 16 °C. The relative humidity ranged from 25% in December and January to 95% in March and April (ANACIM 2015).

### Remote sensing data

Changes in agricultural surface areas of the study zone are estimated quantitatively using satellite image processing. Smart Personal Object Technology (SPOT) satellite images from 1999, with a 10-m resolution, from 2003 with a 5-m resolution and a third one from 2009 with a 2.5-m resolution were obtained from the Astrenium Company. A LANDSAT image from 2014 (30 m) was also downloaded from the Glovis site. The tools used for the remote sensing have given satisfactory results related to the linear analysis of the evolution of Sahel areas (Loum 2012; Ndao 2012; Poncet 1986). The processing of satellite images (SPOT and LANDSAT) with Envi and ArcGIS software associated with global positioning system (GPS) collections for training data has allowed the monitoring of the dynamics of agricultural lands in the study area. The plan for soil sampling was also set up from a photo interpretation of the satellite images.

### The field data

Fieldwork included digging pedologic pits, soil sampling and collecting statistical data from vegetable crops. A stratified method of soil sampling, based on the occupation of the ground, has allowed the setup of 74 soil sampling points in the area of study taking into account farming zones as well as areas for habitat. For each point, soil samples were taken from the following horizons: 0 cm – 10 cm, 10 cm – 20 cm, 20 cm – 40 cm, 40 cm – 60 cm and 60 cm – 80 cm. In each of the four villages (Sébikotane, SébiPonty, Déni Malick and Keur Ndiaye Lo), two pits were dug while respecting the stratification between areas for habits and farming areas (Diack et al. 2017). A description of the soil profiles was followed by a survey with farmers and data collected have allowed to calculate yields for cabbage, carrot, onion and tomato from control plots. The number of farmers surveyed has been defined according to the size of the agricultural population per village. Thus, in Déni Malick and Sébikotane, 50 farmers were chosen in each village, while 15 farmers were surveyed in Keur Ndiaye Lo and Sébiponty, respectively.

### Statistical data analysis from vegetable crop production

As usual, data on vegetable crop production were hardly available. Therefore, surveys conducted with several departments of the Ministry of Agriculture (National Programme of Horticulture, Regional Department of Agriculture) and a non-governmental organisation (NGO) called the Niayes Installation and Economic Development Programme (PADEN) could not allow to collect data from the villages composing the study zone. Surveys were conducted in the Niayes region, with farmers’ representatives from the four villages, on vegetable crop production over the last 15 years.

### The Classification and Regression Tree model

Classification and Regression Tree is a classification method that uses historical data to construct the so-called decision trees. Decision trees are used to classify new data. In order to use CART, it is necessary to know a number of classes a priori. In other words, it is a model of the family of data mining. Data mining is a data analysis technique that allows extracting interesting information, unknown beforehand and potentially useful, from a large-scale database (Han & Kamber 2001). It is composed of a wide range of algorithms with heritages and different strategies (Behrens & Scholten 2007). Two methods of learning mainly set up the modelling process by the approach of data mining: the non-supervised learning methods and the supervised ones. Classification for non-supervised method is characterised by the absence of variables to explain (Besse & Le Gall 2006). Procedure consists of setting up a typology of observed samples into homogeneous classes that best simplify the issues to address. For the supervised learning methods, it is about finding an F function that can explain Y while having an observed X. CART belongs to the set of the supervised learning methods. The principle of functioning of CART is based on the configuration of a studied population based on an optimal rule for classifying the variable to be explained from a combination of explanatory variables. Vulnerability level is a qualitative variable, whereas the explanatory variables are quantitative except for the variable ‘exposure’ which is a qualitative variable. Packages rapart and rapart.plots have allowed calibrating the CART model in the R software. The mathematical formalism of CART starts with the Gini index (Breiman et al. 1984) with:

P(k/p)=1;i(p)=P(k/p)*P(k’/p)avec k≠k’ [Eqn 1]

A *p* node becomes a leaf if i(p) ≤ i_0_ or np ≤ n_0_ with i_0_ and n_0_ values to be determined.

With the R software, a linear regression function (y~ax_1_+ax_2_+… axn, in addition to the matrix of data) will allow to generate the classification tree. In this case, *Y* represents the level of vulnerability and *X* was the explanatory variable of the model CART, which includes the agricultural areas, crops production, incomes, employment and exposure.

### Data processing

**FIGURE 3:**
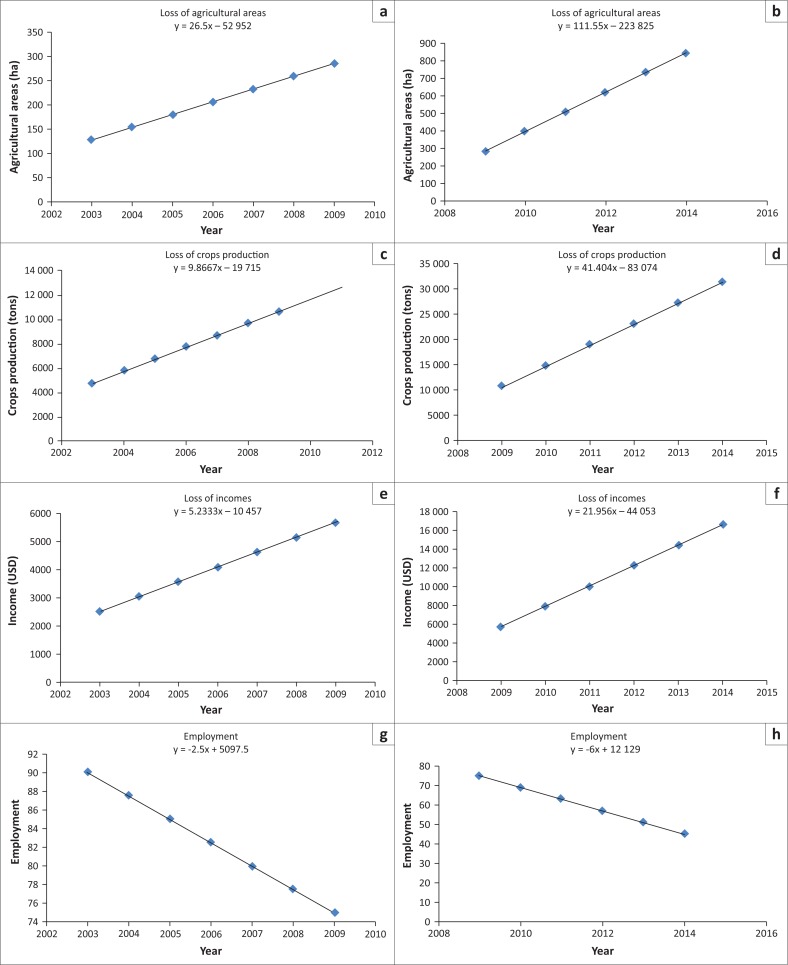
Estimation of the input variables for the Classification and Regression Tree model using linear regression. (a) Loss of agricultural areas 2003–2009, (b) loss of agricultural areas 2009-2014, (c) loss of crop production 2003–2009, (d) loss of crop production 2009–2014, (e) loss of income 2003–2009, (f) loss of income 2009–2014, (g) decrease in employment 2003–2009 and (h) decrease in employment 2009–2014.

Variables of entry for the CART model were obtained only for the years 2003, 2009 and 2014. Satellite image has allowed informing about changes in agricultural areas over these 3 years. After acquisition of statistical data on the agricultural areas, crops production was calculated based on the average yield of horticultural crops in the Niayes region. Statistical data on employment and incomes of the local populations were obtained with socio-economic surveys. The hypothesis of a linear change of the different variables has allowed the building of a matrix of data over the 2003–2014 period (Figure 3) for the calibration of the CART model over R.

## Results

The description of soil profiles from the pits shows different layers with soil depths varying from 36 cm to 124 cm and a mean depth of 79.5 cm. Layers composing the soil profiles have for the most A, AB or BC horizon. Colour of the soils, from the surface down to the deep horizons varies from 2.5 YR (light reddish brown) to 7 YR (greyish brown) to 10 YR (dark greyish brown). The texture ranges from loamy to clayey soil in the soil profile. The soil structure varies from massive, angular to polyhydric status within the soil profile and throughout the four villages. The pH values of the soil ranged between 7 and 8. Salinity is less important with sodium contents of 0.5 Cmol kg^−1^ of soil. The range of organic carbon content was between 5 g kg^−1^ and 25 g kg^−1^. Soil texture is predominantly clayey and then loamy, with a fine fraction content between 60% and 90%. Sandy soils represent about 10% of the texture. Calcium contents ranged from 4 Cmol kg^−1^ to 25 Cmol kg^−1^ of soil. The variability of the magnesium was between 0.5 Cmol kg^−1^ and 14 Cmol kg^−1^ of soil. The Cation Exchange Capacity (CEC), whose values were comprised between 8 Cmol kg^−1^ and 34 Cmol kg^−1^, showed appreciated characteristics of the clayey–humic complex. Data from the soil analyses highlight a good level of soil quality. A description of the soil profiles also attests an existing shallow groundwater of 70 cm depth. Water resources availability and the presence of good quality soils make the Niayes region a suitable area for agriculture. However, the monitoring of the surface areas using satellite image processing shows a loss of the agricultural land from 1999 to 2014. With an area of 2384 ha in 1999, dynamics of agricultural lands show a decrease of -128 ha, -287 ha and -843 ha, respectively, in 2003, 2009 and 2014 from the overall area of study. The main causes of the decrease for the agricultural land were the proximity of the study area with the newly erected city of Diamniadio and the new Blaise Diagne international airport, the consequence of which was the settlement of buildings and industrial establishments in the agricultural land. Socio-economic surveys conducted with local farmers have indicated that the main vegetable crops were produced in the region. Cabbage, carrots, onions and tomatoes cover approximately 95% of the areas used for vegetable crop production. Surveys on crop yields show the importance of cabbage production, with an average yield of 53 t ha^−1^, followed by carrot (35 t ha^−1^), then tomato (26.5 t ha^−1^) and onion (20 t ha^−1^) (Table 1).

**TABLE 1:** Agricultural areas and yields from vegetable crops production in the four villages.

Villages	Crops produced							
	Cabbages		Carrots		Tomatoes		Onions	
	Area (ha)	Yield (t/ha)	Area (ha)	Yield (t/ha)	Area (ha)	Yield (t/ha)	Area (ha)	Yield (t/ha)
Sébikotane	45.00	30.00	25.00	37.50	10.00	22.00	15.00	10.00
SébiPonty	50.00	56.00	20.00	25.00	10.00	27.00	15.00	16.20
Deni Malick	25.00	55.00	25.00	40.00	25.00	25.00	20.00	25.00
Keur Ndiaye Lo	50.00	70.00	15.00	38.00	20.00	32.00	10.00	30.00
Mean	42.50	52.75	21.25	35.13	16.25	26.50	15.00	20.30

*Source*: Direction Sénégalaise de l’Horticulture, 2016, *Recensement de l’horticulture et mise en place d’un système permanent de statistiques horticoles dans la zone des Niayes*, Ministère de l’agriculture et de l’équipement rurald, 51 p and PADEN, 2016, Dakar, Senegal

A comparison between the mean agricultural surface areas per vegetable crop and the dynamics of these areas shows a decrease in the agricultural areas of 126.45 ha for onion and 358.28 ha for cabbages in 2014 (Table 2). The volume of production estimated from an average yield (Table 3) also shows a decrease in production of about 18 900 tons for cabbages, 6292 tons for carrots, 3630 tons for tomatoes and 2566 tons for onion. Such a decrease in the volume of vegetable crops has involved considerable loss of income for the local populations. The purchase price at the farmer’s level varies from 175 Franc –French Community of Africa (FCFA) per kilogram (kg) of onion to 375 FCFA per kg for carrot (Table 4). Surveys conducted with farmers revealed that nearly 90% of the production is sold in the local and national markets and the remaining 10% was intended for self-consumption. Losses of income resulting from the sale of cabbages, carrot, tomato and onion were about $10 176 380, $3 630 000, $1 421 170 and $977 300, respectively (Table 4). With such loss of income resulting from transforming agricultural areas with the profit of habitat, and industrial establishment, local populations become more and more exposed to poverty. Food insecurity risk also becomes real, given the fact that vegetable crop production was primarily intended to satisfy the external demand.

**TABLE 2:** Changes in agricultural farm areas on vegetable crops basis.

Year	Crops produced				
	Cabbages (ha)	Carrots (ha)	Tomatoes (ha)	Onions (ha)	Others (ha)
2003	−54.40	−27.20	−20.80	−19.20	−6.40
2009	−121.98	−60.99	−46.64	−43.05	−14.35
2014	−358.28	−179.14	−136.99	−126.45	−42.15

*Source*: Direction Sénégalaise de l’Horticulture, 2016, *Recensement de l’horticulture et mise en place d’un système permanent de statistiques horticoles dans la zone des Niayes*, Ministère de l’agriculture et de l’équipement rurald, 51 p and PADEN, 2016, Dakar, Senegal

**TABLE 3:** Changes in quantities of production per type of vegetable crops.

Year	Crops produced			
	Cabbages (tons)	Carrots (tons)	Tomatoes (tons)	Onions (tons)
2003	−2869.60	−955.40	−551.20	−389.76
2009	−6434.18	−2142.19	−1235.89	−873.92
2014	−18899.01	−6292.20	−3630.17	−2566.94

*Source*: Direction Sénégalaise de l’Horticulture, 2016, *Recensement de l’horticulture et mise en place d’un système permanent de statistiques horticoles dans la zone des Niayes*, Ministère de l’Agriculture et de l’Equipement rurald, 51 p and PADEN, 2016, Dakar, Senegal

**TABLE 4:** Estimate of income generated per vegetable crop produced.

Crops	Economic parameters					
	Price (FCFA kg^−1^)	Crop production (tons)	Quantity of crops sold (tons)	Quantity of crops sold (kg)	Income (FCFA)*	Income (USD)
Cabbages	350	18 899	17009.10	17 009 100	5 953 185 000	10176384.62
Carrots	375	6292	5662.80	5 662 800	2 123 550 000	3630000.00
Onions	175	3630	3267.00	3 267 000	571 725 000	977307.69
Tomatoes	360	2566	2309.40	2 309 400	831 384 000	1421169.23

* , 1 USD = 500 FCFA.

**FIGURE 4:**
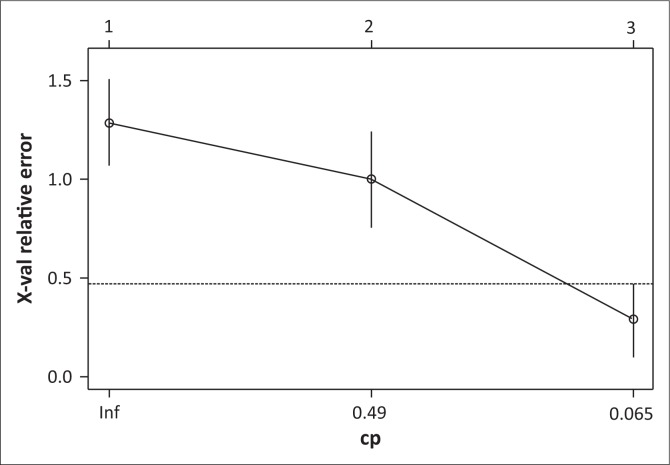
Error rate for the tree generation with the Classification and Regression Tree model.

**FIGURE 5:**
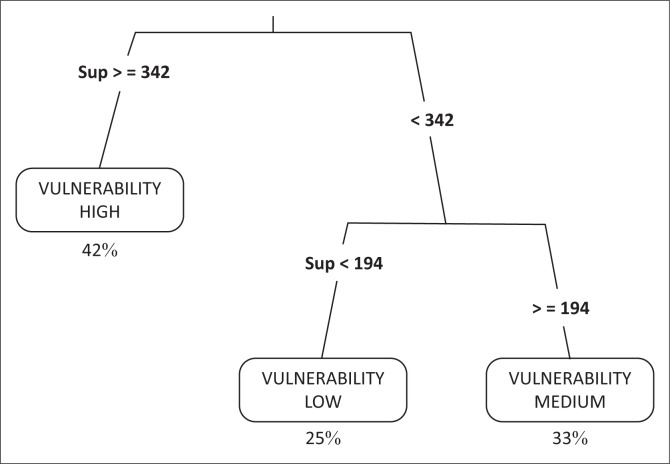
Classification of the level of vulnerability with the Classification and Regression Tree model.

The matrix of data (Table 5) obtained from the linear regression method has allowed the generation of the classification tree. The CART model is calibrated with an error rate of 0.06 (Figure 4). The dynamics of occupation of the land over a decade has shown a level of vulnerability of 42%. Iteration on the last leaves of the tree has shown medium and low levels of vulnerability with 33% and 25%, respectively (Figure 5).

**TABLE 5:** Input variables for the Classification and Regression Tree model.

Year	Level of vulnerability	Agricultural surface areas (ha)	Crop production (tons)	Income(USD)	Employment (%)	Exposure
2003	Low	128.00	4765.96	2527.00	90.0	No
2004	Low	154.00	5786.68	3053.32	87.5	No
2005	Low	180.50	6773.35	3576.65	85.0	No
2006	Medium	207.00	7760.02	4099.98	82.5	Little
2007	Medium	233.50	8746.69	4623.31	80.0	Little
2008	Medium	260.00	9733.36	5146.64	77.5	Little
2009	Medium	287.00	1068.60	5667.00	75.0	Little
2010	High	398.00	14804.00	7856.00	69.0	Yes
2011	High	509.20	18944.40	10051.60	63.0	Yes
2012	High	620.40	23084.80	12247.20	57.0	Yes
2013	High	735.73	27225.20	14442.80	51.0	Yes
2014	High	843.00	31388.00	16645.00	45.0	Yes

## Discussion

In estimating the dynamics of farmlands, tools for space analysis (teledetection and geographical information system [GIS]) have played a major role (Chen et al. 2006). The temporal follow-up of the occupation of the land in the study area, using satellite image processing, has highlighted a regressive change in the farmlands. They went from 1384 ha in 1999 to 1541 ha in 2014, that is to say, a mean reduction rate of 56 ha per year. Urbanisation and implantation of the industrial establishments were the main factors for the reduction of the agricultural areas in the study zone. The urbanisation process was supported by the proximity of the region with the capital city (Dakar) and the installation of the new urban pole of Diamniadio, under the authority of public works. Urban growth of the Dakar region has taken place to the detriment of the farmlands of the Niayes in Pikine and Rufisque (Sy et al. 2014). However, the soil physical and chemical analysis has shown a good agricultural productivity of these lands. Soils of the region have a good quality level with a pH of about 7.5. Salinisation process is less important with a sodium concentration of 0.5 Cmol kg^−1^ of soil, whereas in the humid zone of the Senegal River, the level of concentrations can reach 1.5 Cmol kg^−1^ of soil (Diack et al. 2015). Soil organic matter is also present in acceptable concentrations (2 g kg^−1^) compared to that of the interior region where soil organic matter rarely exceeds 1 g kg^−1^ of soil (Pontanier & Roussel 1998). In addition to the soil quality, water resources are available with aquifers near the soil surface. These agropedologic and hydrologic factors, which are quite favourable, made the Niayes region a suitable zone for vegetable crop production, with yields being able to reach 52 t ha^−1^ for cabbages and 35 t ha^−1^ for carrots. With these assets, agriculture could play a leading role in improving the living conditions of the local population (Direction Senegalaise de l’Horticulture 2016). It contributed to food security and generated not only employment but also income for the local populations. However, the urbanisation process has made the Niayes agricultural region vulnerable and posed real risks of food insecurity, knowing that the greatest contribution of the vegetable crops to the food demand in Senegal comes from that region (Luers et al. 2003). Loss of income resulting from the decrease in agricultural activities exposed the populations, mainly youth and women, to poverty. Agricultural surface areas have decreased at a worrying speed, especially between 2009 and 2014, where 556 ha of agricultural land have been transformed into sites for habitat or industrial areas (Girardin, BocKstaller & Werf 1999). Simultaneous analysis of different agronomic and socio-economic variables using CART has shown a high level of vulnerability of the study zone, which is about 42%. One of the advantages in using CART is the possibility to highlight the explanatory variables by order of importance (Alinovi et al. 2010). Results show that the decrease in agricultural surface areas is the most discriminant one in this study. Over the 12 years of monitoring the dynamics of the agricultural areas (2003–2014), CART presents a high level of vulnerability when decrease in agricultural surface areas is greater than 342 ha. Vulnerability to food insecurity is at medium level when decrease in agricultural surface areas is between 194 ha and 342 ha. Vulnerability level becomes low when reduction of agricultural surface areas is less than 194 ha. In the long run, all agricultural production systems located in the littoral region will be exposed to vulnerability issues (Berry et al. 2006). Risks of food insecurity become more serious because the supply in fruits and vegetables for the interior demand will totally depend on the importation of food substances (Barret 2010).

## Conclusion

A set of tools for space analysis (GIS and remote sensing) and observations made on the physical environment and socio-economic surveys conducted with local populations has allowed to quantify vulnerability indicators in the Niayes agricultural region. Application of the CART model has also allowed to quantify the level of vulnerability of the zone. Findings show the need for preserving the agricultural vocation of the Niayes region. The speed of reduction of the agricultural surface areas has specially increased between 2009 and 2014 with a loss of 65% of these areas. Vegetable crops production, which usually provided means of subsistence to the local populations and served as sources of income, are in the process of disappearing in the Sébikotane, SébiPonty, Déni Malick and Keur Ndiaye Lo villages. Loss of income from the main vegetable crops (cabbages, carrots, tomatoes and onions) estimated based on the decrease of the agricultural lands (1999–2014) is of $16 205 000. Some great public infrastructures, including a turnpike and a new university campus built by the public authority, will accelerate the urbanisation process, making the Niayes agricultural region increasingly vulnerable. In addition, the development of strategies for building resilience in the context of climate change must imperatively take into account scenarios of reduction of the greenhouse gas emission factors. Implantation of industrial companies in the study zone does not participate in the reduction of risks of climate change. Furthermore, the need for reinforcing the resilience of local populations, especially in the Sahelian regions, requires support and extension services for the farmers to promote sustainable agricultural practices. The type of management for the agricultural areas in these regions can be revised by strengthening the potential of storing the soil organic carbon. Agroforestry practices in the Niayes region using an association of fruit trees and vegetable crops provided possibilities of carbon sequestration in the soil and vegetation compartments. Maintenance and improvement of the agricultural production systems could contribute to minimising food insecurity risks to which populations of the sub-Saharan regions are exposed, and mitigate, at the same time, harmful effects of the human activities on climate change.
